# Validation of the Adult Eating Behavior Questionnaire in an Italian Community Sample

**DOI:** 10.3390/nu16060829

**Published:** 2024-03-14

**Authors:** Emanuela S. Gritti, Ludovica Cionti, Federica Cortesi, Alessandro Torelli, Andrea Gambarini, Claudia Hunot-Alexander, Anna L. Ogliari

**Affiliations:** 1 Faculty of Psychology, Milano Bicocca University, 20126 Milan, Italy; 2 Humanistic Studies Department (DISTUM), Carlo Bo University, 61029 Urbino, Italy; 3 Faculty of Psychology, Vita-Salute San Raffaele University, 20132 Milan, Italy; 4 Child in Mind Lab, Vita-Salute San Raffaele University, 20132 Milan, Italy; 5 San Raffaele Hospital, 20132 Milan, Italy; 6 Istituto de Nutricìon Humana, Centro Universitario de Ciencias de la Salud, Universidad de Guadalajara, Salvador Quevedo y Zubieta, Guadalajara 44340, Mexico

**Keywords:** eating, behavior, appetitive traits, adult, validation, questionnaire

## Abstract

(1) Background: Appetitive traits in adults can be measured through the Adult Eating Behavior Questionnaire (AEBQ), a questionnaire adapted from the Child Eating Behavior Questionnaire (CEBQ). The AEBQ has been validated in several countries. The aim of the present study was to explore and validate the factor structure of the Italian version of the AEBQ. Furthermore, convergent validity and correlations between factors and BMI were explored to assess its criterion validity. (2) Methods: Participants (N = 624, mean age of 32.08 ± 14.94 years) completed the AEBQ, the Eating Attitude Test (EAT-40), and the Dutch Eating Behavior Questionnaire (DEBQ). They also self-reported demographic and anthropometric data. A Confirmatory Factor Analysis (CFA) was used to test three different alternative models that emerged in previous validations. (3) Results: The CFA revealed a good model fit (RMSEA = 0.0634, TLI = 0.894, CFI = 0.907) for the 7-factor structure, without the Hunger items, showing a valid and reliable (Cronbach’s α > 0.7) structure. Convergent and divergent validity of the AEBQ yielded favorable results, and relationships between the AEBQ and BMI factors revealed that the Food Approach traits were positively associated with BMI. (4) Conclusions: Finally, this study provides initial support for the use of the AEBQ as a valid and reliable tool to measure a wide range of appetitive traits in the adult Italian population.

## 1. Introduction

Several dysfunctional eating behaviors are thought to be determined by both the environment and individual appetitive traits [1]. Such traits are defined as a set of persistent predispositions and behavioral tendencies toward food and eating opportunities [2,3]. Appetitive traits interact with environmental factors, but their genetic component is widely recognized [1]. This intrinsic feature of appetitive traits has led over the years to perceive them as a basically stable component over time [4], similar to a “basic” individual feature of one’s relationship with food that can determine eating choices across the lifespan. Importantly, several appetitive traits measured in childhood have demonstrated associations with unfavorable outcomes, such as higher adiposity, that can predispose to problematic conditions, such as rapid weight gain in infancy and obesity in childhood and later in life [5]. The correct assessment of such dispositions towards eating is fundamental, as well as considering their influence on dysfunctional eating behaviors, for which they may constitute a risk factor [2,4,6,7,8].

The field of psychological assessment is currently permeated with an ongoing debate, as to whether categorical or dimensional classification systems are the best way to conceptualize psychopathology and behaviors [9]. This topic is currently a subject of debate within the domain of eating behaviors as well [9,10]. Certainly, the finding that a dimensional model provides the best fit to the data does not automatically rule out the possibility that some aspects of the latent structure of eating behaviors may be better conceptualized categorically [11,12,13,14]. However, growing scientific evidence indicates that dimensional models may be more suitable for explaining the complex structure of eating disorders, including high levels of comorbidity and variability within diagnostic categories [10,15]. The dimensional model views individual differences as variations in the degree of the target psychological characteristic, allowing the assessment of eating behaviors not only in the clinical population, but also in the general population [9,16,17]. As such, contemporary measures of eating behavior disturbances have taken a dimensional stance; for example, the Three-factor Eating Questionnaire (TFEQ) [18] and the Dutch Eating Behavior Questionnaire (DEBQ) [19] have been used as measures of appetitive traits in adults.

The TFEQ measures three appetitive traits: Cognitive Restraint, Disinhibition, and Hunger. The DEBQ measures Emotional Eating, Restrained Eating, and External Eating. In this framework, the more recent Adult Eating Behavior Questionnaire (AEBQ) can be employed to measure a greater number of appetitive traits [3] usefully expanding the description of individual appetitive profiles. The AEBQ was adapted from the Child Eating Behavior Questionnaire (CEBQ) [17], changing the response format from CEBQ parent report to AEBQ self-report [3,6]. Similarly, to the CEBQ, the AEBQ measures eight appetitive traits [3]; however, seven dimensions have been most commonly found to be reliable [8,20].

Appetitive traits are measured using the AEBQ by two different subscales: four traits are related to the “Food Approach” dimension (Hunger, Food Responsiveness, Emotional Overeating, Enjoyment of Food) and four to the “Food Avoidance” (Satiety Responsiveness, Emotional Undereating, Food Fussiness, Slowness in Eating) [3,4,20]. Specifically, (1) Hunger refers to the internal and physical sensation of appetite [20] and (2) Food Responsiveness may characterize a maladaptive heightened susceptibility to eating in response to external food cues [17,20]. Excessive overeating behavior is measured by the (3) Enjoyment of Food, where the focus is on pleasure rather than necessity [20]. The Emotional Eating subscales break down into two subscales and refer to eating due to negative emotions: (4) Emotional Overeating and (5) Emotional Undereating [3]. These traits refer to the consumption of food related to negative emotional experiences [20]. The (6) Satiety Responsiveness scale measures the ability to regulate food intake in relation to the feeling of satiety [5]. (7) Slowness in Eating is used to describe a person’s own perception of how quickly they eat. (8) Food Fussiness refers to an extremely selective attitude towards the range of foods that the individual normally eats [17].

The Adult Eating Behavior Questionnaire has been validated in adult populations from the United Kingdom [3], Australia [8], China [6], Mexico [21], and Canada [20]. The AEBQ was also validated in adolescent populations from the United Kingdom [22], Poland [4], and Portugal [23]. To date, the validity of the AEBQ has not been tested on Italian-speaking populations. The adaptation and development of culturally appropriate tools are necessary to better understand the etiology of appetitive traits within each country and to study their application in the context of adaptive and maladaptive eating patterns.

### The Present Study

The aim of this study was the adaptation and validation of the AEBQ into Italian in a representative general population sample. In particular, this research project aims to describe the psychometric properties of the AEBQ for the identification of the best factor structure by testing different alternative models. In addition, the internal consistency and the construct validity of the subscales of the AEBQ were assessed. The association between AEBQ factors and self-reported BMI was also investigated. Based on previous validations mentioned above, positive correlations between the Food Approach scales and BMI, and negative correlations between Food Avoidance scales and BMI are expected [3,8,20].

The second aim was to test the construct (i.e., convergent) validity of the AEBQ between the specific subscales of the AEBQ and the DEBQ subscales. According to results from Hunot and colleagues [22], positive correlations are expected between the AEBQ Food Approach subscales and the DEBQ External Eating and Emotional Eating subscales, as well as positive correlations between the AEBQ and DEBQ Restrained Eating scale.

Finally, comparisons were conducted for Emotional Overeating and Emotional Undereating traits among different populations: (1) population at risk/not at risk of developing an eating disorder (see Statistical Analyses section for more details); (2) BMI (Underweight, Normal Weight, Overweight); and (3) gender (male vs. female). According to the literature, higher levels of Emotional Eating are expected for the population at risk of developing an eating disorder than for the not-at-risk population [6,7]. Furthermore, the evidence suggests that females have greater emotional eating behaviors than males [4]. Such differences represent one of the core aspects of ED; as such, the label “high-” vs. “low-risk” population derives from a higher expression of under- or overeating behaviors vs. a lower expression. In addition, the prevalence of ED is greater in the female population [24]. To date, the study of eating behaviors in adults has usually focused on appetitive traits related to the risk of obesity and/or dysfunctional eating patterns [3,6,8]. This study aims to expand the investigation of appetitive traits among underweight individuals. According to the results of Jacob and colleagues, a significant difference in Emotional Eating is expected among different BMI groups [20]. Specifically, overweight individuals should show higher levels of Emotional Overeating and lower levels of Emotional Undereating, and a logical extension to this should be a reversed pattern for underweight or low BMI individuals.

## 2. Materials and Methods

### 2.1. Participants

The sample of participants was recruited through social media via two researchers’ personal accounts (WhatsApp (version 2.22.2.15, WhatsApp Inc., Mountain View, CA, USA), Instagram (version 230.0, Instagram, LLC, San Francisco, CA, USA)). The participants completed an anonymous online survey with voluntary participation: no financial reward was provided for filling in the questionnaire. The data were collected between March and April 2022. The data collection period did not correspond with the vacancy period (Easter); therefore, we have no reason to believe that there were any influences on appetitive traits. The only inclusion criterion was being over 18 years of age. The participants were asked if they had previously suffered from any eating disorders, but such a variable was not considered an exclusion criterion. The participants provided written informed consent before entering the study. In total, 624 compilations were collected. The research was conducted in agreement with the guidelines of the Declaration of Helsinki and was approved by the Vita-Salute San Raffaele University Institutional Review Board (Eat 2020).

#### Italian Translation of the Adult Eating Behavior Questionnaire (AEBQ)

The transcultural adaptation of the Italian version of the Adult Eating Behavior Questionnaire (AEBQ) was conducted by the research team members according to established international guidelines [25,26] and World Health Organization directions. Two researchers, who were fluent in both Italian and English, translated the 35 items of the AEBQ into Italian. Researchers with expertise in eating behavior traits oversaw the Italian translation to a revision. Then, the back translation was made by an external collaborator who was a professional translator living in an English-speaking country [25]. Finally, the translated version was administered to a small group of Italian native speakers who were not fluent in English and naïve on the psychological dimensions measured by the AEBQ. No further linguistic changes have been made to the Italian scale, due to the correspondence of the back-translation with the original version of the AEBQ.

### 2.2. Measures

The measures were administered through an online survey: the sections are listed in detail below.

#### 2.2.1. Sociodemographic, Anthropometrics and Body Satisfaction

The following demographic information for the participants was collected: age, gender, highest level of education completed, and profession. Measurements of body weight (kg) and height (m) were self-reported. To comply with the participant’s potential willingness to not disclose weight and height, the option to skip these questions was available in the survey. Using these data, the BMI (Body Mass Index) was obtained (kg/m^2^). Finally, they were asked to express their satisfaction with their body weight, indicating, in the event of a negative response, what their ideal weight was. Finally, participants were asked to report whether they had ever suffered from any eating disorder (ED); the possible answers were: “Yes”, “No”, and “I don’t know”.

#### 2.2.2. Adult Eating Behavior Questionnaire (AEBQ)

Appetitive traits were assessed using the AEBQ questionnaire. The AEBQ is a 35-item self-report questionnaire that comprises four subscales of “Food Approach” and four subscales of “Food Avoidance”. “Food Approach” includes the following: Hunger (H; 5 items, e.g., “I often feel hungry”); Food Responsiveness (FR; 4 items, e.g., “I am always thinking about food”); Emotional Overeating (EOE; 5 items, e.g., “I eat more when I’m upset”); and Enjoyment of Food (EF; 3 items, e.g., “I enjoy eating”). “Food Avoidance” includes the following: Satiety Responsiveness (SR; 4 items, e.g., “I get full easily”); Emotional Undereating (EUE; 5 items, e.g., “I eat less when I’m worried”); Food Fussiness (FF; 5 items, e.g., “I refuse new foods at first”); and Slowness in Eating (SE; 4 items, e.g., “I eat slowly”). Item responses were recorded on a 5-point Likert scale ranging from “Strongly Disagree” (1) to “Strongly Agree” (5) [3]. In accordance with the original version of the AEBQ, three items of Food Fussiness and one item of Slowness in Eating were characterized by a reverse score. Mean scores were calculated for each subscale.

#### 2.2.3. Eating Attitude Test (EAT-40)

The Eating Attitude Test (EAT-40) is a self-report questionnaire that evaluates the potential presence of dysfunctional eating behaviors; in particular, the risk of developing an eating disorder, with particular attention to the symptoms of Anorexia Nervosa [27]. For the present study, the Italian validation of the EAT-40 was used [28]. The scale is a 40-item self-report questionnaire (e.g., “Feel extremely guilty after eating”) that uses a 6-point Likert scale ranging from “Always” (1) to “Never” (6). The scoring of items 1, 18, 19, 23, and 39 is 6 = 3 points; 5 = 2 points; 4 = 1 point; 3, 2 or 1 = 0 points. The remaining items have the following scoring algorithm: 1 = 2 points; 2 = 2 points; 3 = 1 point; and 4, 5 or 6 = 0 points. The total score is given by the sum of all item scores; a total score greater than 30 is an indicator of “high risk” of eating disorders [27].

#### 2.2.4. Dutch Eating Behavior Questionnaire (DEBQ)

The Dutch Eating Behavior Questionnaire (DEBQ) is a self-report questionnaire for assessing eating behaviors through 33 items. The DEBQ contains three subscales: Emotional Eating (13 items, e.g., “Do you have a desire to eat when you are irritated?”); External Eating (10 items, e.g., “If food smells and looks good, do you eat more than usual?”); Restrained Eating (10 items, e.g., “Do you try to eat less at mealtimes than you would like to eat?”) [19]. Responses to the items are evaluated using a 5-point Likert scale ranging from “Never” (1) to “Very often” (5). Item 31 has a reverse score. Mean scores were calculated for each subscale. For this project, the Italian validation of the DEBQ was used [29].

### 2.3. Statistical Analysis

Descriptive statistics were run for socio-demographic, anthropometric, and body satisfaction characteristics, divided by gender. Subsequently, the normality of the items of the AEBQ scale was verified (Table 1).

To test the factor structure of the AEBQ within the Italian population, Confirmatory Factor Analysis (CFA) was conducted, and three previously suggested models were tested [3,8,20,23,30]. “Model 1” is the original 8-factor model (35 items) of the AEBQ. “Model 2” is the 7-factor solution (35 items), with Hunger and Food Responsiveness items loading onto the same factor. This factor structure has been identified in a Principal Component Analysis by Hunot and colleagues [3]. “Model 3” is a 7-factor model (30 items) that excludes the Hunger subscale [3,4,8,22,30]. The goodness of fit statistics, including the Comparative Fit Index (CFI), Tucker–Lewis Index (TLI), and Root Mean Square Error of Approximation (RMSEA), were obtained for each model and interpreted according to established guidelines. According to Hu and Bentler [31], values ≥ 0.90 for CFI and TLI, and ≤0.08 for RMSEA were considered an adequate fit [31]. The internal reliability of each AEBQ factor was assessed using Cronbach’s α; values greater than 0.70 are indicators of good reliability [32].

Relationships between AEBQ scales and BMI were examined. Subsequently, the correlations between the Food Approach subscales and the AEBQ Food Avoidance subscales were calculated. After verifying the normality of the AEBQ and DEBQ subscales [33] and evaluating the internal reliability of the DEBQ factors [32], convergent validity was tested using Pearson’s correlation [34].

The comparison between the emotional eating subscales (EOE and EUE) of the AEBQ was then carried out via one-way ANOVA. The sample was divided into “at risk” (>30, EAT-40 cut-off) and “not at risk” (<30, EAT-40 cut-off) subsamples based on the score of EAT-40 [27], specifically comparing the averages of each emotional eating dimensions, EOE and EUE. Then, mean EOE and EUE for male and female subsamples [35] were compared. After verifying the normality of the EOE and EUE subscales in populations, Test F and T-test [36,37] were conducted. Finally, BMI group differences (Underweight, <18.5; Normal Weight, 18.5 < BMI > 24; and Overweight, >24) were observed for EOE and EUE [38] with one-way ANOVA. After verifying the normality of the EOE and EUE variables in the three different subsamples and homogeneity of variances (Levene Test), Test F and T-test [37] were conducted.

## 3. Results

### 3.1. Descriptive Statistics

The distribution of items for all subscales measured moderately approximated a normal distribution [39].

The sample consisted mainly of female subjects (68.1%). The mean age was 32.08 ± 14.94 years (range 18–83). Descriptive statistics for the (n = 624) filtered by gender are presented in Table 2. In total, 10 participants provided missing data for weight and height; for this reason, they were not included in the analysis of BMI group differences.

### 3.2. Factor Structure Analysis

The AEBQ factor structure was tested through CFA. Table 3 shows the fit indices of the three-factor models of AEBQ identified in previous validation [3,8,23]. “Model 3” showed the best model fit (RMSEA = 0.0634, TLI = 0.894, CFI = 0.907) [32].

The internal reliability analysis of the AEBQ showed a Cronbach’s α greater than 0.80 (range 0.829–0.920) for all AEBQ factors, except for Satiety Responsiveness (0.690) (Table 1). The item–rest correlation always returned values greater than 0.40 [40].

### 3.3. Intercorrelations among AEBQ Subscales and BMI

As expected, positive and significant intercorrelations were observed between all Food Approach subscales and between most Food Avoidance subscales. Along similar lines, negative correlations were observed within the Food Approach subscales and Food Avoidance subscales. Small negative correlations were observed between BMI and the Food Avoidance subscales, except for Food Fussiness. Conversely, small positive correlations were obtained between BMI and Food Approach subscales, except for Food Responsiveness (Table 4).

### 3.4. Intercorrelations among AEBQ and DEBQ Subscales

Table 5 shows the correlations between the three subscales of the DEBQ and the seven factors of the AEBQ identified in the present Italian sample. The correlations between the AEBQ Food Approach subscales and the DEBQ subscales were positive and statistically significant. No significant correlation was found between Enjoyment of Food and Restrained Eating. The correlations between the AEBQ Food Avoidance subscales and the Emotional Eating and External Eating subscales were negative, but not all statistically significant. The DEBQ Restrained Eating subscale showed a significant, albeit low, positive correlation with Satiety Responsiveness. Furthermore, the Restrained Eating subscale did not show significant correlations with the other AEBQ Food Avoidance subscales, except for Satiety Responsiveness.

### 3.5. Mean Comparisons between EOE and EUE Subscales Scores

Results from Table 6 show the comparison between mean EOE and EUE subscale scores among different subsamples (Student’s *t*-test). The results showed statistically significant differences, with the at-risk group reporting higher levels of EOE. No significant difference for EUE was observed.

### 3.6. Mean Comparisons among BMI Groups on EOE and EUE

Applying analysis of variance (ANOVA) for the samples divided into three BMI groups (Underweight, Normal Weight, and Overweight) showed significant differences between them. Individuals with overweight or obesity had higher scores for EOE and lower scores for EUE, as compared to underweight individuals. The comparison between Normal Weight and Overweight revealed higher scores in overweight people for EOE and no significant differences for EUE. The comparison between EOE and EUE mean scores for underweight and normal-weight individuals did not show a significant difference. Finally, the results revealed statistically significant differences for gender, as females reported higher levels of EOE and EUE.

## 4. Discussion

The present study aimed to explore the structure, consistency, and validity of the AEBQ in a fairly large sample of the Italian general population. Findings from the CFA support a seven-factor structure of the Italian version of the questionnaire, with a total of 30 items that measure three Food Approach and four Food Avoidance behaviors. The CFA supported the factor structure of “Model 3” as the best fit for the Italian population. Among the three alternative models [3,8,20,23,30], only the seven-factor solution, without the Hunger items, showed adequate fit indices.

Introducing the Adult Eating Behavior Questionnaire (AEBQ) in the Italian clinical and research context represents an important contribution to the field, adding to existing questionnaires that assess eating behaviors across different stages of the life course. By tracking appetitive traits from infancy (Baby Eating Behavior Questionnaire, BEBQ) [1] and childhood (CEBQ) into adulthood (AEBQ), researchers are able to gain a more comprehensive understanding of the relationship between appetitive traits and weight throughout a person’s life. Such a standpoint is also crucial to identify and monitor more closely for risk factors for eating disorders (EDs) [7], which are complex disturbances with a multifactorial etiology [38]. In this line of reasoning, a question about the presence of eating disorders was added to the form; this question was added in order to evaluate the subjective perception of problematic eating behaviors during the entire span of life. Also, importantly, the increased prevalence of problematic eating behaviors during and after the COVID-19 pandemic [41] was evidenced in self-reported answers, as seen in the present sample. The self-report question was about problematic eating behaviors and was meant to investigate the presence of different behaviors (from no carbs at dinner to DCA symptomatology).

Appetitive traits result from a complex combination of genetic predisposition and biological factors, which are shaped by environmental factors such as family environment, cultural context, and peers [5]. Given all these influences, the development of culturally appropriate tools to measure these traits across cultures is necessary [23,42].

### 4.1. Construct Validity of the Italian AEBQ and Association with BMI

The AEBQ Food Approach subscales were correlated to each other, as expected [3]. Similarly, positive intercorrelations were also present among the Food Avoidance subscales [3]. Positive significant correlations were observed between the three Food Approach subscales (FR, EOE, and EF), and between the Food Avoidance subscales (SE, EUE, and SR) of the AEBQ, whereas generally negative and significant correlations were observed between the subscales measuring ostensibly different dimensions [3]. No significant associations were found for the AEBQ Food Fussiness subscale (FF), except for a negative correlation between FF and EF, as already observed in the literature [43]. This might be due to the opposite descriptions of the two factors: food selectivity versus enjoyment of food.

The relationships between appetitive traits and BMI observed in the Italian adult sample were similar to findings from the literature [3]. Higher BMI was consistently positively associated with Food Approach and negatively associated with Food Avoidance subscales [3], indicating that these subscales capture dimensions of appetite. FF was unrelated to BMI, further supporting the idea that this scale reflects a qualitatively different Food Avoidance trait [8], which, in adults, is less common than in children, and directed towards a much smaller number of foods [3,44,45,46]. The FR dimension, reflecting possible restrained eating in some individuals [8], had a small and non-significant negative correlation with BMI, similar to what was found in the Australian validation of the AEBQ [8]. The small negative associations of FR with BMI could reflect adult behavior in response to physical hunger. This response does not necessarily lead to food ingestion thanks to self-regulation abilities and might therefore reduce the expression of this trait and its association with greater calorie intake and higher BMI [8,22].

Alternatively, the small non-significant negative correlation between the FR factor and BMI similarly could represent an eating behavior [47] more typical of the female population—which represents the majority of this sample—i.e., a less reactivity to food [8]. It seems plausible that actively restricting food intake may result in more frequent feelings of greater responsiveness to the sight and smell of palatable foods [22].

Finally, the interpretation of this discrepant correlation finding between FR and BMI is complicated by the fact that BMI was self-reported and therefore less reliable than objectively measured anthropometrics [8]. As such, further tests of these hypotheses are needed to better understand how this scale should be interpreted; in this line of reasoning, associations between FR and measures of dietary restraint should be investigated.

The construct validity of the AEBQ was supported by significant correlations between specific subscales of the AEBQ and their counterparts of the DEBQ [22], as well as by negative correlations with putatively opposite constructs. This result provides evidence supporting the validity of the AEBQ as a reliable tool to assess eating behaviors among Italian adults [23,30]. There was good convergent validity between the AEBQ Food Approach subscales and the DEBQ Emotional Eating and External Eating scales, which emerges from the statistically significant positive correlations found (*p* < 0.01). The significant positive correlation between the subscales had already been similarly observed in an English adolescent population [22]. Mainly in line with the expectations, small, negative, and significant correlations between the AEBQ Food Avoidance scales and External Eating were found (*p* < 0.01), except for EUE.

A strong significant and positive correlation between Emotional Eating (DEBQ) and EOE (AEBQ) was found, together with a significant negative correlation between Emotional Eating (DEBQ) and EUE (AEBQ). This further supports the convergent validity of the AEBQ [2] and its capacity to measure the different directions of the Emotional Eating construct [3].

Unlike the results obtained in the English adolescent population [22], the correlations between the Food Avoidance subscales (AEBQ), except for Satiety Responsiveness, and the DEBQ Restrained Eating factor were negligible, indicating good divergent validity. In fact, the term “Restrained Eating” refers to limiting food intake to control body weight [29]; although the AEBQ can be used to tailor weight loss interventions [22], no AEBQ factor has been designed to measure intentional eating behavior for weight control [3,22]. Thus, the absence of correlations between Food Avoidance and Restrained Eating (DEBQ) supports the divergent validity of the AEBQ, as noted by He et al. [6]. A negligible but significant negative correlation was observed, similar to what was found in the English population [22].

A somewhat unexpected pattern of correlations among AEBQ SR, FR, and EOE subscales with the DEBQ Restrained Eating subscale was found. These relationships were positive and significant: such a result has to be interpreted considering several aspects. As far as found in the literature, restrained eaters control their food intake. They may pay more attention to food and diet-related cues. In fact, restrained eaters display an attentional bias toward food cues as well as selective memory for them [48]. When faced with appealing food cues, individuals who practice restraint in their eating habits tend to exhibit heightened responsiveness to food stimuli. However, this pattern can be reversed when restrained eaters are exposed to food cues labeled as “healthy”. It has been observed that restrained eaters are more likely to eat the food labeled as “healthy” and often to eat more of it [48].

Food deprivation can stimulate overeating [49]. Several paradigms, such as the cycles of dieting and overeating, have demonstrated how prolonged periods of restriction can lead to an increase in food consumption even after the restriction has ended, indicating a persistent behavioral tendency towards food. These findings contribute to this somewhat unexpected association [50,51].

### 4.2. Emotional Eating between Different Groups: Risk of Eating Disorders, BMI, Gender

Importantly, numerous studies have shown that emotions play a significant role in regulating food intake [52]. As such, Emotional Eating, measured using the AEBQ, has been linked to the development of EDs [3,52,53,54]. However, most of these studies have focused only on EOE, which is characterized by overconsumption of food due to negative emotions, and have neglected EUE [52,55]. By incorporating both traits of Emotional Eating, in a dimensional framework, the AEBQ provides a more comprehensive understanding of how emotions influence food intake, particularly in individuals who may be at risk of developing an ED.

We analyzed the role of both EOE and EUE in relation to the risk of presenting an eating disorder using a comparison between groups at different risks of developing an ED. Firstly, the presence of a difference between the risk and non-risk groups of EDs for EOE e EUE was investigated. Notably, to our knowledge, previous studies had never considered Emotional Eating in its two opposite dimensions, and this is therefore a novel approach to this issue. As shown in the current study, individuals at risk of EDs reported higher levels of EOE than people not at risk, coherently with the findings from previous studies [52]. On the other hand, the literature suggests that individuals with EUE were at a higher risk of ED [52,56], but the present study showed no significant differences between the two groups. The absence of significant findings in our study could be due to the great difference between the size of the two groups, with the at-risk group being so much smaller. In addition, our sample was drawn from the general population, where the actual presence of ED diagnosis is putatively low or undiagnosed.

The comparison between normal weight and overweight subgroups revealed higher scores in overweight people for EOE, confirming previous results [20] and no significant differences for EUE. Individuals with overweight reported higher scores for EOE and lower scores for EUE, as compared to underweight individuals. According to Frayn et al. [57] and Ozier et al. [58], overweight people have greater difficulties in using effective coping skills when dealing with negative emotions. As a result, they tend to turn to emotional eating more frequently. The lack of significant difference between EOE and EUE mean scores for underweight and normal-weight individuals could be due to the significant difference in the size of the two groups, where the largest group was normal weight. In this regard, the uniqueness of this study is that it has allowed for comparison not only between EOE and EUE contrasting overweight and normal-weight individuals [20] but also considered underweight subjects.

A significant difference was observed in this study between male and female participants in terms of the emotional appetitive traits of the AEBQ, with higher levels for both EOE and EUE in females. This was expected considering similar previous findings [4,20], and at least two lines of interpretations could be given. Firstly, the higher scores of Emotional Eating in female participants might be due to the higher tendency of women to self-attribute characteristics of “vulnerability”, such as interpersonal dependence in explicit measures (e.g., self-reports) [59]. Secondarily, as previous studies demonstrated, females report higher levels of neuroticism and more difficulties in hunger control than males [60,61,62,63]. Although the latter finding might be due to an overreporting of problematic aspects, as well as to some gender bias present in personality self-reports [64], it might also be the case that Emotional Eating is one possible response to discomfort [52,53,54] that is more present in women.

### 4.3. Limitations and Future Directions

There were some limitations in the research conducted. Firstly, the sample analyzed mostly consisted of young adult females, as in other validations of AEBQ [8,20,30], mainly from Northern Italy. Thus, future studies should involve a larger population, divided by the area of origin, to consider possible differences. Secondly, the research relied solely on online questionnaires, which may have limited the outreach to those who have access to technology and education [3]. Moreover, the measures, especially weight and height were self-reported, leading to possible bias like BMI calculation [3,6,8]. Participants may have felt the need to respond in a socially desirable manner due to awareness of the links between eating behaviors and weight, according to the literature [3,6,8]. Obtaining more objective measurements in the future is recommended to enhance the precision of assessing the measured constructs/dimensions. Notably, however, self-report questionnaires are widely used across research on nonclinical and clinical populations and can be beneficial to let the subjectivity of the respondent emerge, allowing, at the same time, rapid and inexpensive screening.

Furthermore, the present study was conducted on a sample from the general population, and as such, it was not possible to investigate the expression of the target appetitive traits using the AEBQ in a clinical setting. While studies in nonclinical samples are crucial for comprehending the mechanisms underlying eating behaviors and their potential subclinical manifestations, future investigations should employ the AEBQ to better elucidate the contribution of appetite traits to maladaptive eating patterns.

In conclusion, the AEBQ could be used to develop personalized interventions aimed at helping individuals manage their weight by addressing their unique appetitive trait responses [3]. By developing a personalized appetitive profile, it is possible to identify, especially in cases requiring intervention, the traits where the individual faces the greatest difficulties [21]. This could allow for a tailored intervention but also prevention of the person and their specific complexity, an approach that is particularly appropriate for individuals with Eds, as well as to avoid therapeutic alliance ruptures in treatment [65].

## 5. Conclusions

The present research indicates that the Italian version of the AEBQ is a valid and reliable tool for measuring eating behavior traits within the Italian population and stilling a behavior profile. This questionnaire holds significance as the first tool in Italy to measure seven appetitive traits (four more than the TFEQ and DEBQ previously used), thus enabling us to assess a more comprehensive appetite profile. It is recommended that further validation against diverse clinical populations and measurements be conducted to establish its comprehensive utility in assessing a broad range of eating behaviors primarily related to appetite. Furthermore, the AEBQ is suggested to be a valuable tool for evaluating eating behaviors in clinical interventions for eating disorders and obesity treatment and prevention.

## Figures and Tables

**Table 1:** Descriptive statistics: normality and internal consistency.

Variable	Skewness	Kurtosis	Cronbach’s Alpha
FR_AEBQ	0.30	−0.28	0.720
EF_AEBQ	−0.73	0.13	0.765
EOE_AEBQ	0.42	−0.76	0.920
FF_AEBQ	0.78	−0.01	0.877
SE_AEBQ	0.43	−0.53	0.848
EUE_AEBQ	0.01	−0.89	0.914
SR_AEBQ	0.54	0.30	0.690
TOT_Fappr_AEBQ	0.21	−0.28	0.857
TOT_Favoid_AEBQ	0.28	0.32	0.828
EMO.E_DEBQ	0.42	−0.58	0.960
EXT.E_DEBQ	0.73	−0.05	0.853
RE_DEBQ	0.09	−0.11	0.919
TOT_DEBQ	0.46	−0.06	0.927
TOT_EAT40	1.77	3.24	0.904
BMI	1.60	5.19	

FR_AEBQ, “Food Responsiveness”; EOE_AEBQ, “Emotional Overeating”; EF_AEBQ, “Enjoyment of Food”; EUE_AEBQ, “Emotional Undereating”; FF_AEBQ, “Food Fussiness”; SE_AEBQ, “Slowness in Eating”; SR_AEBQ, “Satiety Responsiveness”. TOT_FApp_AEBQ, “Food Approach”; TOT_Favo_AEBQ, “Food Avoidance”. EMO.E_DEBQ, “Emotional Eating of DEBQ”; EXT.E_DEBQ, “External Eating of DEBQ”; RE_DEBQ, “Restrained Eating of DEBQ; TOT_DEBQ, “DEBQ Total”; TOT_EAT40, EAT-40 Total; BMI, “Body Mass Index”.

**Table 2:** Demographic characteristics of the sample and descriptive statistics by group.

	Sample (n = 624)	Females (n = 425)	Males (n = 194)
	n (%)	n (%)	n (%)
Gender			
Female	425 (68.1%)		
Male	194 (31.1%)		
Not specified	5 (0.8%)		
Weight satisfaction?			
Yes	345 (55.3%)	174 (40.9%)	103 (53.09%)
No	279 (44.7%)	251 (59.1%)	91 (46.91%)
Eating Disorder?			
Yes	95 (15.2%)	85 (20%)	10 (5.16%)
No	450 (72.2%)	275 (64.7%)	171 (88.14%)
Don’t know	79 (12.7%)	65 (15.3%)	13 (6.7%)
	Sample (n = 624)	Females (n = 425)	Males (n = 194)
	Ȳ ± s (range)	Ȳ ± s (range)	Ȳ ± s (range)
Age	32.08 ± 14.94 (18–83)	31.54 ± 14.58 (18–79)	33.44 ± 15.76 (18–83)
BMI ^a^	22.43 ± 3.74 (15.06–47.75)	21.86 ± 3.93 (15.06–47.75)	23.66 ± 2.93 (17.54–34.72)
	Sample (n = 624)	F (n = 425)	M (n = 194)
	Ȳ ± s	Ȳ ± s	Ȳ ± s
FR	2.89 ± 0.82	2.97 ± 0.86 ^b^	2.74 ± 0.70
EOE	2.67 ± 1.16	2.82 ±1.20 ^b^	2.34 ± 0.97
EF	4.10 ± 0.71	4.11 ± 0.72	4.04 ± 0.68
EUE	2.88 ± 1.13	2.95 ± 1.14 ^b^	2.75 ± 1.10
FF	2.11 ± 0.91	2.13 ± 0.91	2.06 ± 0.87
SE	2.49 ± 1.01	2.56 ± 1.05 ^b^	2.34 ± 0.91
SR	2.38 ± 0.78	2.52 ± 0.80 ^b^	2.10 ± 0.62
FApp_AEBQ	3.22 ± 0.68	3.30 ± 0.71 ^b^	3.04 ± 0.57
FAvo_AEBQ	2.47 ± 0.60	2.54 ± 0.61 ^b^	2.31 ± 0.54
TOT_EAT-40	13.48 ± 9.79	15.03 ± 10.68 ^b^	10.16 ± 6.44
RE_DEBQ	2.51 ± 0.95	2.62 ± 1.01 ^b^	2.28 ± 0.76
EMO.E_DEBQ	2.37 ± 1.04	2.56 ± 1.09 ^b^	1.96 ± 0.79
EXT.E_DEBQ	3.28 ± 0.71	3.33 ± 0.71 ^b^	3.16 ± 0.69

FR, “Food Responsiveness”; EOE, “Emotional Overeating”; EF, “Enjoyment of Food”; EUE, “Emotional Undereating”; FF, “Food Fussiness”; SE, “Slowness in Eating”; SR, “Satiety Responsiveness”. FApp_AEBQ, “Food Approach”; Favo_AEBQ, “Food Avoidance”. EMO.E_DEBQ, “Emotional Eating of DEBQ”; EXT.E_DEBQ, “External Eating of DEBQ”; RE_DEBQ, “Restrained Eating of DEBQ”. ^a^ = for this variable, the sample was 614 (417 F, 192 M). ^b^ = statistically significant difference between groups (F > M). Ȳ ± s, “Mean ± Standard Deviation”.

**Table 3:** Confirmatory Factor Analysis results.

Model	Items	Factors	CFI	TLI	RMSEA	ꭕ^2^ (*df*)	ꭕ^2^/*df*	AIC	BIC
1	35	8 (H and FR load on separate factors)	0.894	0.882	0.0602	1734 (532)	3.25940	58415	59005
2	35	7 (H and FR load on combined factor)	0.890	0.878	0.0610	1792 (539)	3.32468	58459	59018
3	30	7 (H items deleted)	0.907	0.894	0.0634	1346 (384)	3.50521	49152	49645

FR, “Food Responsiveness”; H, “Hunger”; CFI, “Comparative Fixed Index”; TLI, “Tucker–Lewis Index”; RMSEA, “Root Mean Square Error of Approximation”. ꭕ^2^/*df*, Chi-square standard.

**Table 4:** Correlations between subscales and Food Approach and Food Avoidance subscales.

		Food Approach Scale			Food Avoidance Scale						
		FR	EOE	EF	EUE	FF	SE	SR	F.Appr	F.Avo	BMI
	FR	1	0.317 **	0.568 **	−0.066	−0.110 *	−0.149 **	−0.231 **	0.779 **	−0.213 **	−0.009
Food Approach scale	EOE		1	0.240 **	−0.478 **	0.035	−0.101 *	−0.118 *	0.777 **	−0.295 **	0.235 **
	EF			1	−0.214 **	−0.264 **	−0.112 *	−0.384 **	0.711 **	−0.375 **	0.107 *
	EUE				1	0.066	0.138 **	0.368 **	−0.371 **	0.679 **	−0.195 **
Food Avoidance scale	FF					1	0.015	0.270 **	−0.116 *	0.507 **	−0.034
	SE						1	0.272	−0.156 **	0.585 **	−0.158 **
	SR							1	−0.293 **	0.721 **	−0.220 **
	F.Appr								1	−0.383 **	0.167 **
	F.Avo									1	−0.245 **

FR, “Food Responsiveness”; EOE, “Emotional Overeating”; EF, “Enjoyment of Food”; EUE, “Emotional Undereating”; FF, “Food Fussiness”; SE, “Slowness in Eating”; SR, “Satiety Responsiveness”; F.Appr, “Food Approach”; F.Avo, “Food Avoidance”. *p* * < 0.05 = correlation is significant at the 0.05 level (2 tailed). *p* ** < 0.001 = correlation is significant at the 0.01 level (2 tailed). Pearson’s correlation was used for normally distributed mean scores.

**Table 5:** Construct validity.

		Food Approach Scale			Food Avoidance Scale			
		FR	EOE	EF	EUE	FF	SE	SR
	Restrained Eating	0.136 **	0.207 **	−0.049	−0.005	−0.023	−0.019	0.170 **
DEBQ	Emotional Eating	0.444 **	0.790 **	0.300 **	−0.401 **	−0.015	−0.066	−0.155 **
	External Eating	0.627 **	0.229 **	0.383 **	−0.025	−0.118 *	−0.154 **	−0.206 **

FR, “Food Responsiveness”; EOE, “Emotional Overeating”; EF, “Enjoyment of Food”; EUE, “Emotional Undereating”; FF, “Food Fussiness”; SE, “Slowness in Eating”; SR, “Satiety Responsiveness”. *p* * < 0.05 = Correlation is significant at the 0.05 level (2 tailed). *p* ** < 0.001 = correlation is significant at the 0.01 level (2 tailed). Pearson’s correlation was used for normally distributed mean scores.

**Table 6:** Emotional Overeating and Emotional Undereating mean differences by eating disorder risk, BMI group, and gender.

Factors	ED Risk			BMI Group			Gender			
	Risk (n = 53)	No Risk (n = 571)	*p*-Value	Underweight (n = 52)	Normal Weight (n = 552)	Overweight (n = 113)	*p*-Value	Female (n = 425)	Male (n = 194)	*p*-Value
Emotional Overeating	3.13 ±1.38	2.63 ± 1.13	0.003	2.47 ± 1.08 †	2.57 ± 1.15 *	3.14 ± 1.15	0.001	2.82 ±1.20	2.34 ± 0.97	0.00001
Emotional Undereating	2.89 ± 1.31	2.88 ± 1.11	0.98	3.26 ± 1.21”	2.89 ± 1.12	2.68 ± 1.10	0.009	2.95 ± 1.14	2.75 ± 1.10	0.04

* vs. “Overweight” *p* < 0.001; † vs. “Overweight” *p* < 0.001; ” vs. “Overweight” *p* = 0.003. ED, “Eating Disorder”.

## Data Availability

The data that support the findings of this study are available on request from the corresponding authors, L.C. and F.C. The data are not publicly available due to confidentially agreement with study participants.
